# Evaluating teaching effectiveness in nursing education:An Iranian perspective

**DOI:** 10.1186/1472-6920-5-29

**Published:** 2005-07-27

**Authors:** Mahvash Salsali

**Affiliations:** 1 Associate Professor Faculty of Nursing, University of Tehran, Iran

## Abstract

**Background:**

The main objective of this study was to determine the perceptions of Iranian nurse educators and students regarding the evaluation of teaching effectiveness in university-based programs.

**Methods:**

An exploratory descriptive design was employed. 143 nurse educators in nursing faculties from the three universities in Tehran, 40 undergraduate, and 30 graduate students from Tehran University composed the study sample. In addition, deans from the three nursing faculties were interviewed. A researcher-developed questionnaire was used to determine the perceptions of both faculty and students about evaluating the teaching effectiveness of nurse educators, and an interview guide was employed to elicit the views of deans of faculties of nursing regarding evaluation policies and procedures. Data were analyzed using parametric and nonparametric statistics to identify similarities and differences in perceptions within the Iranian nurse educator group and the student group, and between these two groups of respondents.

**Results:**

While faculty evaluation has always been a major part of university based nursing programs, faculty evaluation must be approached more analytically, objectively, and comprehensively to ensure that all nursing educators receive the fairest treatment possible and that the teaching-learning process is enhanced.

**Conclusion:**

Educators and students stressed that systematic and continuous evaluation as well as staff development should be the primary goals for the faculty evaluation process. The ultimate goals is the improvement of teaching by nurse educators.

## Background

The aims of nursing education principally center on the transmission of nursing knowledge, and assisting nursing students to acquire the necessary skills and attitudes associated with nursing practice. As with professional preparation generally, nursing education encompasses the three domains of learning, the cognitive, the affective, and the psychomotor. One way to enhance nursing education is to evaluate the effectiveness of teaching in nursing education programs. An interest in evaluating teaching effectiveness has increased over time and acceptance of the need to evaluate teaching has continued to grow. Defining what we mean by effective teaching, however, presents a challenge to the profession.

Teaching is an art and it should be judged for the passion and beauty of the performance and the meaningfulness of the message conveyed [1]. Teaching is a complex and demanding activity that involves mastery of content, classroom control, techniques of organization, and command of teaching skills. Teaching consists not only of instruction, but also of the systematic promotion of learning by whatever means [2-5]. The profiles of effective teachers are as diverse as the students they teach. Still, the best teachers do share several characteristics. In Qualities of Effective Teachers, Stronge (2002) synthesizes research to identify specific teacher behaviors that contribute to student achievement. Rather than look at outside factors like demographics, district leadership, and state mandates, Stronge focuses specifically on what teachers can control-their own preparation, personality, and practices. Fitzpatrick (2004) comments that though it is important to develop more comprehensible means to measure effectiveness, it is equally important to recognize that one may be able to truly measure the art of teaching in conventional ways.

While these global perspectives are important they do not identify many of the specifics that are associated with effective teaching in nursing education and the means that could or should be used to assess teaching effectiveness in nurse preparation programs. These specifics include: determining what are the effective teaching skills, what beliefs about the teaching and learning process educators and their students hold, what criteria should be used to assess teaching effectiveness, who should evaluate the various aspects of teaching, and what other important elements should and do guide the assessment of effective teaching in nursing education. Identifying these components is necessary for educators to improve their teaching and, ultimately, for helping aspiring nurses acquire the beliefs, the skills, and the knowledge that are needed in nursing practice. Indeed, evaluation of teaching is central to providing feedback to educators, and for providing reliable and valid information for the tenure and promotion process.

In the past two decades as economic realities and accountability requirements have affected higher education, the evaluation of educators in nursing education programs has become an important issue. Documenting teaching effectiveness in nursing education is essential to demonstrating nursing education's accountability to the profession and to the public it serves. For the teaching of prospective nurses to remain a dynamic activity, regular evaluation is vital [2,6]; it is equally important for nurse educators to develop their teaching by systematic evaluation. The evaluation of teaching facilitates attainment of several important objectives: to improve the quality of teaching, to assist faculty to evaluate their own teaching, to fulfill the criteria of the academic institution, to improve accountability in education, and to identify the content areas for faculty development programs [7,8]. Arthur Jr et al (2003) mention that the most appropriate criterion for assessing teaching effectiveness is a function of the goal of evaluation. Evaluation of teaching is important in the teaching-learning process. The review of evaluation data can identify areas of effectiveness, as well as problem areas in teaching. If nurse educators are to grow as effective teachers they will need knowledge of the teaching and learning process as well as an understanding of the criteria used to assess their effectiveness as teachers.

Nursing education in Iran has undergone much change since the first training schools for nurses were opened in the 1940s. This change has been associated with corresponding changes in societal values, in the health care system, and in the political environment. Having become accepted as a respectable occupation within Iran nursing has now turned its attention to professional respectability. This professional respectability has become almost synonymous with academic respectability and the positioning of nursing education as a complex academic undertaking.

At present, the nursing programs in Iran offer a four year baccalaureate in nursing accredited by the High Council of Medical Education of the Ministry of Health and Medical Education. The curriculum is designed as a *community oriented nursing program *with the philosophy of *health for all *and employs *primary health care *(PHC) strategies. Also, the concepts of primary, secondary, and tertiary prevention are integrated throughout the nursing curriculum. The four year nursing education program consists of three years of theoretical education in which courses are pursued in faculties of nursing and one year of clinical practice in hospitals. Following completion of their nursing education program, nurses participate in the nursing comprehensive examination under the supervision of the Ministry of Health and Medical Education. These examinations are the equivalent of the North American RN examinations and serve as nursing licensing /registration examinations. Success on these examinations allows nurses to practice nursing in the country. Nursing graduates of BSc degree programs may enter MSc programs if they pass the entrance examination. At present there are 148 nursing education centers offering the BSc degree, 12 centers offering the MSc degree, and three centers offering the PhD degree in nursing, with plans underway for a fourth [9].

This study was the first research endeavor into teaching effectiveness evaluation in the faculties of nursing in Tehran. The purpose of this paper is to report on the perceptions of nursing educators and students in Iran with respect to actual and preferred evaluation methods – who evaluates and how – including identification of their beliefs about the teaching-learning process, criteria for evaluating teaching effectiveness, and the impact of evaluation elements on teaching-learning outcomes. The findings of this study provide some direction on where we might begin to understand and to improve nursing education in Iran.

### Research questions

The research questions of the study addressed the following seven areas: (a) actual and preferred methods of evaluating the teaching effectiveness of Iranian nurse educators, including who does the evaluation; (b) the beliefs of Iranian nurse educators and students about the teaching and learning process; (c) the importance of selected criteria in evaluating the teaching effectiveness of nurse educators; (d) the influence of selected elements in evaluating the teaching effectiveness of nurse educators; (e) the degree of agreement and differences between nurse educators and nursing students in the actual and preferred methods of evaluating teaching effectiveness, beliefs about teaching and learning, evaluation criteria and evaluation elements; (f) the relationship of personal and professional variables to the perceptions of nurse educators and students concerning evaluation methods, beliefs about teaching and learning, evaluation criteria, and evaluation elements; and (g) the perceptions of deans of nursing concerning policies and procedures related to nurse educator evaluation.

#### Research design and methods

An exploratory descriptive design was employed. A questionnaire was developed to determine the perceptions of the two main categories of participants, faculty and students. The items relate to methods of evaluation, evaluation practices, evaluators, beliefs about teaching and learning, criteria for evaluating teaching effectiveness, evaluation elements, and selected personal and professional variables. Content validity, a test-retest procedure, and a pilot study were used to test and to increase the validity and reliability of the questionnaire. Personal interviews were conducted to elicit information from the deans of nursing in three large Faculties of Nursing in Tehran regarding evaluation policies and procedures.

### Research setting and study participants

Three nursing faculties of the universities in Tehran comprised the setting for this study. These universities are controlled by the Ministry of Health and Medical Education. Approximately 200 full-time faculty members (100%) from the three universities, as well as 36 (100%) graduate students and a stratified random sample of 80 (10%) undergraduate students from one of these universities were invited to participate in the study. In total, 143 educators, 40 undergraduate students, and 30 graduate students returned questionnaires providing a response rate of 71% for educators, 50% for undergraduate students, and 83% for graduate students. Each of the deans of nursing from the three universities was interviewed. Students were selected from one university for reasons of convenience and the uniformity of entrance standards, curriculum, and education qualifications across these faculties of nursing. Comparisons between students and educators were done using the three faculty groups combined following an ANOVA test which identified no statistically significant differences among them.

The majority of faculty members in this study were female (92%), between the ages of 40 and 49 (51%), married (77%), prepared at the master's degree level (89%), teaching entirely in the baccalaureate program (65%), involved in both classroom and clinical education (94%), and had 10 years or more of teaching experience (69%) and five years or more of clinical experience (63%). Twenty percent were involved in the supervision of research and a large majority provided evidence of scholarly productivity (89%). The majority of student respondents were female (80%), between the ages of 20 and 29 years (74%) and single (61%), with slightly more than half in the baccalaureate program (57%) and the remainder at master's level.

### Questionnaire construction

The questionnaire was developed by the researcher after a review of the literature pertaining to both nursing and education, and consultation with professionals with expertise related to this subject. The questionnaire had six. In *Section I*, participants were asked to provide information about their personal and professional background. *Section II *had two parts. In part *A*, participants were requested to rate nurse educator evaluators in terms of their perceptions of the involvement which each evaluator had (actual) and should have (preferred) in the evaluation of teaching effectiveness (A five point Likert scale from *very limited *to *very great involvement*). In part *B of Section II *participants were requested to rate evaluators according to perceived specific input, process, and output criteria in both actual and preferred situations. In *Section III*, participants were requested to rate evaluation practices in terms of their perceptions of the perceived use of each practice (actual) and the preferred use in evaluating teaching effectiveness (A five point Likert scale from *very limited *to *very great use*). *Section IV*, participants were asked to rate beliefs about teaching and learning in terms of their perceptions of the degree of agreement with each belief in the evaluation of teaching effectiveness (A five point Likert scale from *strongly disagree *to *strongly agree*). In *Section V*, participants were requested to rate criteria in terms of their perceptions of the importance of each criterion in evaluating teaching effectiveness. In *Section VI *of the questionnaire, participants were requested to rate evaluation elements in terms of their perceptions of the importance of each element (A five point Likert scale from *very limited *to *very great importance. *Upper case likert scale items contained a sixth option don't know). Pilot testing of the questionnaire was conducted with a convenience sample of nurse colleagues from Alberta, Canada and Tehran, Iran.

## Results

Quantitative data were analyzed using parametric and nonparametric statistics to identify perceptions and differences that exist within the Iranian nurse educator group and the student group, and between these two groups of respondents. Content analysis was used to compare and contrast the recorded views of the deans of nursing regarding evaluation policies and procedures. The study findings are presented in six sections: (a) actual and preferred evaluators, (b) actual and preferred evaluation practices, (c) beliefs about the teaching and learning process, (d) evaluation criteria, (e) evaluation elements and (f) deans' perspectives.

### Actual and preferred evaluators

Regarding the question of who was and who should be involved in evaluating teaching effectiveness, educators and students rated five categories of evaluators: administrators, educators, students, peers, and heads of groups (i.e., heads of undergraduate program areas, including medical-surgical, psychiatric, pediatric, health, and administration). Educators' perceptions reveal that students were ranked as being the most frequent source of actual evaluation data (with "moderate involvement"), whereas students in this study perceived that educators themselves were the most frequent source of these data (also, with "moderate involvement"). Both groups agreed that the second, third and fourth ranked evaluators were heads of groups, administrators and peers. Educators ranked themselves fifth, as having "very limited involvement"; in marked contrast, the students felt that they had the least amount of involvement in this process.

On the matter of preferred evaluators, educators viewed self-evaluation as their first choice ("great involvement"), with heads of groups ("moderate involvement") and peers ("some involvement") being their second and third choices. Students agreed with educators that the greatest involvement in nurse educator evaluation should be by educators themselves ("great involvement") with students ranked second (also "great involvement") and heads of groups ranked third (with "moderate involvement"). The degree of actual and preferred involvement of administrators received the same ranking by educators and students. Both groups saw the administrators as the middle group (third) for actual involvement, and preferred that their involvement be the lowest of the five groups.

With regard to the types of evaluators related to the inputs, the process and the outputs of teaching, the greatest consensus among educators for perceptions concerning actual evaluators of input criteria was for heads of groups, while preference was for self evaluation. Students perceived that inputs were evaluated primarily by educators themselves while their preference was for heads of groups and students to do this type of evaluation. For evaluating the process of teaching, the greatest degree of consensus among educators for actual evaluators was for heads of groups, while they preferred to have self evaluation. Among students, two-thirds perceived that educators themselves were the actual evaluators while the students preferred to have students as the primary evaluators of the process of teaching. For evaluating the outputs of teaching, the greatest consensus among educators, with respect to actual evaluation, existed for educators themselves. This was also their preference. Students perceived that educators themselves were the actual evaluators of output, while they preferred to have both student evaluation and evaluation by educators themselves.

### Actual and preferred evaluation practices

On the question of how teaching effectiveness is being evaluated and how it should be evaluated, educators and students rated five categories of practices: performance observation, rating scales, student achievement, teacher tests and self-appraisal. Performance observation, student achievement and rating scales were the most common current evaluation practices as perceived by educators and by students. All three had ratings, on a scale from 1 to 5, between 2.0 (some use) and 3.0 (moderate use). The two groups of respondents agreed that teacher tests and self appraisal were the least-used evaluation practices (between very limited use and some use).

With respect to preferences, educators preferred between great use (4.0) and very great use (5.0) of two of the five practices: self-appraisal and student achievement, and between moderate use and great use of the remaining three. Students placed four practices as having great to very great use: student achievement, teacher tests, rating scales, and self-appraisal, with the remaining practice, performance observation, as having moderate to great use. It is important to note that the means for each of these five practices increased from the actual to the preferred situation (highly significant statistically) for both groups, indicating that the educators and the students were in favor of more emphasis being placed on each practice.

### Beliefs about the teaching-learning process

Participants rated 14 belief statements about teaching and learning in terms of their degree of agreement with each belief statement. "Instructor's role is to facilitate students learning," "respect for students' abilities and experiences," and "instructor should help students choose and develop their own directions for learning," were ranked (in order) significantly higher by educators. "Respect for students' abilities and experiences," "the role of instructor in organizing the content and sequence of learning based on students' needs," and "the instructor should measure teaching effectiveness by assessing changes in students' attitudes and behaviors," were ranked (in order) significantly higher by students.

A factor analysis on the 14 belief statements identified four factors: (a) learning-centered values (including items: the instructor should focus on what is sure, reliable, and lasting., the instructor's role is to facilitate student learning., the instructor should focus on intellectual development., and " the instructor should promote active student participation in deciding what is to be learned and how), (b) teaching-centered values (including items: students are good sources of ideas for improving teaching and learning, the instructor should show each student that her/his abilities and experiences are respected and valued., and the instructor should help students choose and develop their own directions for learning), (c) pedagogical values (including items: the instructor's role is to evaluate students' achievement and assign grades., the instructor should make the decisions about what is to be taught, when, and how, the instructor should be mainly a transmitter of knowledge in the classroom, and the instructor should develop a systematic plan for the course and stick to it)., and (d) andragogical values (including items: the instructor should organize the content and sequence of learning activities based on students' needs, the instructor should measure teaching effectiveness by assessing changes in students' attitudes and behaviours., and the instructor should inspire students to create their own learning activities and materials rather than always provide them." Further analyses revealed that the learning-centered beliefs and teaching-centered beliefs were those most commonly held by educators, and teaching-centered beliefs and andragogical beliefs those most commonly held by students.

### Evaluation criteria

**Figure 1:**
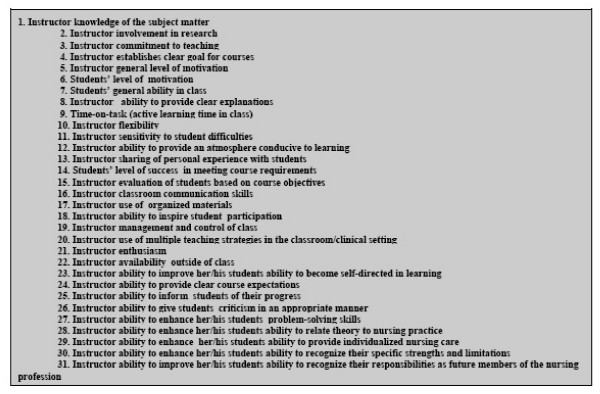
Evaluation Criteria*. * Evaluation criteria are the components that may influence the basis on which a judgment is made, especially input criteria (e.g., student characteristics, teacher characteristics, course characteristics), process criteria (e.g., classroom atmosphere, teacher behaviour), and output criteria (e.g., student achievement) that take into account the context of the teaching.

Study participants rated 31 potential criteria for their importance in evaluating teaching effectiveness (Figure 1). The results indicated that educators' and students' responses were quite similar for various criteria, although there were differences in order. Most of these items such as instructor motivation, clear explanation by instructor, instructor knowledge of the subject matter, instructor commitment to teaching, management and control of class, and student motivation were rated between of great and very great importance by both groups of respondents, and the remainder rated as of least or moderate importance.

A factor analysis was used to determine whether different evaluation criteria could be grouped together. A four-factor solution seemed most suitable: (a) inputs in the teaching-learning process containing five items which represented teaching inputs, such as, "instructor knowledge of subject matter," and "instructor establishes clear goals for courses"; (b) instructor helping behavior, containing 11 items such as, "instructor sensitivity to student difficulties," and "instructor availability outside of class"; (c) instructor teaching behavior: student engagement, containing eight items such as, "instructor ability to inspire student participation," and "instructor ability to enhance her/his students' ability to provide individualized nursing care"; and (d) instructor teaching strategies, containing seven items, including, "instructor ability to provide clear explanations," and "instructor sharing of personal experience with students." These last three factors all contained items which seemed to represent process-product criteria in evaluating teaching effectiveness. Inputs in the teaching-learning process was the first choice, "instructor teaching behavior: student engagement" the second choice, "instructor teaching strategies" third, and "instructor helping behavior" fourth as perceived both by educators and students. There were no significant differences between educators' and students' perceptions for these four categories of evaluation criteria.

### Evaluation elements

**Figure 2:**
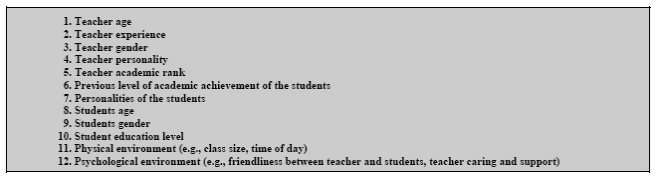
Evaluation Elements*. * Evaluation elements are the components that may influence the basis on which a judgment is made. The components include teacher characteristics, student characteristics, physical environment, and psychological environment.

The two categories of respondents rated the importance which they felt each of 12 elements – "teacher personality," "teacher age," "student personality," and "psychological environment (e.g., friendliness between teacher and students)," etc. – should have in influencing the outcomes of teaching (Figure 2). Educators and students shared similar perceptions concerning the importance of these elements although the rank orders were not identical. Educators rated between "of great importance" and "very great importance" the following items, in rank order: "teacher personality," "psychological environment," "teacher experiences," "student personalities," "physical environment," and "teacher academic rank." Students assigned the same high rating to "teacher experiences," "psychological environment," "teacher academic rank," "teacher personality," and "physical environment."

### Deans' views

The deans of nursing indicated that there were advantages and disadvantages associated with the educator evaluation system that was currently in place. They also emphasized the importance of educator evaluation and of evaluation outcomes, of having these evaluations done by different individuals, of having a systematic and continuous evaluation process and of using different approaches to evaluating teaching effectiveness. They stressed that staff development should be the ultimate goal for the faculty evaluation process. Overall, they recognized that there were shortcomings to the educator evaluation system in use in their faculty and expressed a desire to improve teaching effectiveness within the faculty.

## Discussion

The study has shed some light on the complex process of effective teaching in nursing and on how the assessment of teaching effectiveness might be done. The major findings are as follows:

(1) Although faculty prefers self evaluation and students prefer student evaluation, both reported limited use of multiple evaluators. With the exception of educators' views that students should be less involved in the evaluation of teaching, both groups of respondents preferred greater involvement for the five groups of evaluators: educator (self) evaluation, evaluation by head of group, peer evaluation, administrator evaluation, and student evaluation. There is general agreement in the literature that students, peers and self do not supply sufficient evaluative information on their own but that together they provide a comprehensive evaluation [10,7,12]. Also the use of student ratings of teaching effectiveness is enhanced by an understanding of the nature of the underlying dimensions [13].

(2) Although performance observation, student achievement and rating scales were the most common current evaluation practices, as perceived by the respondents, the two groups reported somewhat limited use of these three practices, and especially of teacher tests and self-appraisal. It was significant that both educators and students expressed a desire for more use of all five evaluation practices. The literature on the efficacy of some of these evaluation practices is equivocal. For example, self-appraisal is seen to have the disadvantages of subjectivity whereas the process of self-appraisal is seen to enable individuals to develop reflection skills, and to enhance understanding of one's own strengths and weaknesses thereby helping them become more self-aware and objective in judging their own performance [14-18]. The use of student behaviors and achievement as criteria of effective teaching has its own challenges. Some of the literature cautions against the positivist approach to the assessment of what are seen to be highly subjective process and outcomes [15,19]. Nevertheless, performance observation is recommended because numerous studies have shown positive relationships of various teacher behaviors – such as effective questioning and answering of questions, giving feedback and use of learning objectives – with student outcomes [20,21]. Student learning is widely believed to be a good measure of teaching effectiveness. Unfortunately, measures of student achievements are not as ideal as they may first appear. Factors such as initial level of student knowledge, student motivation, and student ability are beyond the educator's control yet can strongly affect the amount of student learning [10]. Input from students, for example student ratings, is also recognized as an essential component of a comprehensive system for evaluating teaching effectiveness [10,22]. In general this body of literature supports the use of multiple evaluation practices, with the argument that use of only one method produces a distorted and one-sided picture.

(3) Educators and students were in agreement on the importance of teaching-centered beliefs, but educators also perceived learning-centered beliefs as important whereas for students' andragogical beliefs were more important. The literature underlines the importance of instructor beliefs about the teaching and learning process in affecting the outcomes of teaching [23,24]. Also evident in the findings of recent research is that different teaching styles have success with different students: there appears to be no single best teaching style. Some students have been found to do better with a highly-structured teaching style (teaching-centered) whereas others seem to do better in less-structured settings (learning-centered) (Sinclair, 1987). Related to the outcomes of this study, the adult-learning perspective – andragogy – is deemed important by Knowles (1984) and by Fuszard (1995). It was perhaps understandable that nursing students in the current study placed such high importance on this perspective. On the other hand, the pedagogical perspective – as contrasted with the andragogical – was given low importance ratings by both respondent groups in the current research. This is in keeping with the contentions of Allen (1990) and of Diekelmann (1991 & 1988) who recommend alternate pedagogies for nursing education.

(4) Educators and students reported that most of the criteria for evaluating the inputs, the process, and the outputs of teaching were of "great" or "very great" importance. Also, they shared the same perceptions regarding the importance of the four categories of criteria used in the current study that were ranked in the following order: inputs into the teaching/learning process, instructor teaching behavior: student engagement, instructor teaching strategies, and instructor helping behavior. These findings are supported by the literature. For example, Elliot et al (2000); Biggs (1999); Kirschling et al (1995); Money (1992); O'Neill (1988); Dunkin (1987); and Gracas, Feldens, & Duncan (1986) demonstrate that teacher behaviors such as knowledge of subject-matter and instructor commitment (inputs to the teaching/learning process), the ability to inspire interest, instructor communication skills, and instructor use of organized material (student engagement variables), instructor flexibility and instructor ability to provide clear explanations (instructor teaching strategies), along with instructor enthusiasm, teacher-student relationship, and instructor ability to give students criticism in an appropriate manner (instructor helping behaviors) are all related to effective teaching.

(5) Important elements for evaluating teaching effectiveness were consistent with several studies reviewed in the literature which showed that instructor personality, instructor experience, and the psychological environment were important variables in teaching effectiveness as perceived by educators and by students [14,25,26]. In contrast with the literature, students' and educators' perceptions in this study showed that teacher academic rank was viewed as an important variable for evaluating teaching effectiveness.

(6) The deans of the three faculties of nursing in Tehran were aware of shortcomings of their instructor evaluation system and they expressed the desire to improve teaching effectiveness in their respective faculties. They also emphasized the importance of evaluation and evaluation results, and the need to have these evaluations completed by different individuals. They stressed that systematic and continuous evaluation as well as staff development should be an ultimate goal for the faculty evaluation process.

The author believes that the nature of competence in nursing education is considerably more complex than can be captured in observable, measurable behaviors. However, there are a variety of reasons as to why we need to evaluate teaching effectiveness and the way we teach nursing students in all types of nursing programs: (a) the health care system is not fully meeting societal needs; (b) the student population in nursing programs is changing; (c) the need for caring health professionals has never been more apparent; and (d) proponents of the current curriculum revolution are calling for education models that educate rather than train, that are interactive rather than passive, and that emphasize understanding of principles rather than the lockstep execution of procedures. Thus, the ultimate goal in nursing education should be to provide the best quality educational experiences for all students. Each aspect of a nursing education program should contribute toward accomplishing that goal.

For an evaluation system to contribute to this goal, it must promote the professional improvement of each faculty member and, at the same time, provide information sufficient to identify teaching deficiencies. Educators generally resent being reviewed through a process they view as punitive, and administrators become frustrated with evaluative procedures that have a negative impact on individual faculty members and on faculty climate. Valentine (1992) contends that implementing an evaluation system that improves personnel performance and removes incompetent educators without creating a climate of mistrust and malcontent is one of the most elusive tasks in education. Such a system must also reward competent performance.

## Conclusion

The nursing profession seems to be increasingly concerned with evaluation as part of its accountability. However, the literature indicates that little attention has been paid to the topic of nursing educator evaluation and evaluating teaching effectiveness in Iran as well as in North America and other parts of the world. The evaluation of teaching effectiveness is a complex process and will never be an easy task nor perceived to be a totally fair endeavor for any nursing department. The advantages and limitations inherent in any evaluation system are intensified by the variety of roles and responsibilities assumed by nurse educators. It is most important that a representative and, ideally, comprehensive picture of each educator's performance be obtained from relevant sources. Data collected must be based on criteria, if the evaluation is to have meaning [27,28]. The development of a comprehensive faculty evaluation system that has as one of its important objectives to distinguish good from superior performance can challenge and stimulate all members of faculty to strive for meaningful accomplishments that are both rewarding to the individuals being evaluated, and recognized as important in the administrative decision making process. It is also important that we continue to develop better and more comprehensive measures of teaching effectiveness.

## Pre-publication history

The pre-publication history for this paper can be accessed here:


